# Gastric transcatheter chemoembolization can resolve advanced gastric cancer presenting with obstruction

**DOI:** 10.3389/fsurg.2022.1004064

**Published:** 2022-10-20

**Authors:** Dong Peng, Bin Zhang, Chao Yuan, Yue Tong, Wei Zhang

**Affiliations:** Department of Gastrointestinal Surgery, The First Affiliated Hospital of Chongqing Medical University, Chongqing, China

**Keywords:** gastric cancer, obstruction, gastric transcatheter chemoembolization, outcome, survival

## Abstract

**Background:**

Gastric transcatheter chemoembolization (GTC) is an interventional minimal invasive method, which has never been mentioned in the previous literature for advanced gastric cancer with obstruction. The purpose of this study was to evaluate its safety and efficacy in treating advanced gastric cancer with obstruction.

**Methods:**

Advanced gastric cancer patients with obstruction who underwent GTC were retrospectively analysed from June 2017 to January 2020. Baseline information, peri-intervention data, and post-intervention follow-up information were collected. Clinical data obtained before and after the GTC were compared, and the survival of all patients was analysed.

**Result:**

Forty-Two patients were included in this study. 42 (100%) patients achieved technical success, and 22 (52.4%) achieved clinical success. The median time of the GTC was 83 (30.0–180.0) minutes, and the median time of hospitalization after GTC was 3 (1–6) days. One patient experienced abdominal pain during and after GTC. Twenty (47.6%) of the 42 patients underwent gastrectomy after intervention. The pre-intervention gastric outlet obstruction scoring system (GOOSS) was 1 (0–1) and the post-intervention GOOSS was 2 (0–3) (*p* = 0.000 < 0.05). The median follow-up time was 9.5 (3–35) months, and the overall survival time was 14 months. In the univariate survival analysis, a significant difference was observed between patients who did or did not undergo radical gastrectomy after GTC (*p* = 0.014 < 0.05).

**Conclusions:**

GTC is a safe and effective treatment, and furthermore, it could be an alternative method in treating advanced gastric cancer with obstruction.

## Introduction

Gastric cancer ranks the third in cancer-related mortality and is one of the most lethal malignancies in the world (1). Gastric obstruction often occurs in patients diagnosed with advanced gastric cancer, and may cause abdominal pain, nausea, vomiting, malnutrition and some related metabolic diseases, which affects the quality of life of patients (2, 3). Radical resection is not an optimized solution for advanced gastric cancer, however, the appropriate method for removing the obstruction is the main urgent problem that must be solved (4).

Gastrojejunostomy (GJJ) is a routine surgical treatment for GOO that usually relieves the symptoms of the obstruction. However, many complications have been observed after GJJ, such as an unsatisfactory improvement in oral eating, duodenal reflux, biliary vomiting, and bleeding caused by chyme contacting the tumour surface (5). Although the surgery is relatively simple, the incidence of postoperative complications (13%–55%) and the mortality rate (2%–36%) are high (6, 7). With the continuous development of minimally invasive technology, laparoscopic surgery has begun to be promoted and applied in gastric cancer with obstruction. In general, the use of minimally invasive surgery represents an alternative option at treatment centres with extensive experience in laparoscopic surgery.

In recent years, endoscopic stents have been used to treat obstruction caused by tumours. Compared with surgery, stent implantation by endoscopy has more advantages. For example, patients only require light anaesthesia, their discomfort during treatment is less than surgery, and the endoscopy substantially decreases the time to diet and the length of the hospital stay (8). However, many shortcomings have been noted. There are some complications, including a failure of stent placement, stent displacement, tumour growth along the stent, gastric bleeding, and gastric perforation (9).

Lipiodol transcatheter chemoembolization has been very successful in liver tumors, and the size of tumors decreased obviously after chemoembolization (10–12). However, only transcatheter chemotherapy for gastric cancer has been reported (13), chemoembolization has never been mentioned in the previous literature. In this study, we will review gastric transcatheter chemoembolization (GTC) in treating advanced gastric cancer with obstruction and evaluate its safety and effectiveness.

## Materials and methods

### Patients

We retrospectively analysed patients who were diagnosed with advanced gastric cancer complicated with obstruction in a clinical centre from June 2017 to January 2020. The inclusion criteria were (1) patients who were diagnosed with advanced gastric cancer with obstruction according to gastroscopy and computed tomography (CT) examinations; (2) confirmed as gastric cancer by the pathology; and (3) patients who underwent GTC. The exclusion criteria were patients with an obstruction caused by tumours in the pancreas, bile duct, duodenum and other tissues.

The study was approved by local Ethics Committee, and informed consent was obtained from all patients.

### GTC procedures

**Figure 1:**
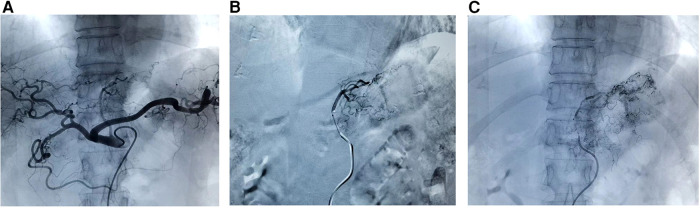
The procedures of GTC. (**A**) Contrast medium injected into celiac trunk to show blood supply; (**B**) superselected into the left gastric artery; (**C**) oxaliplatin, docetaxel and lipiodol mixed with oxaliplatin injected sequentially. GTC, gastric transcatheter chemoembolization.

Seldinger method was used to insert a vascular sheath through the right femoral artery. A 5F angiographic catheter (RLG or RH TPYE, Terumo, Tokyo, Japan) was placed in the celiac trunk, and the contrast medium was injected to show the blood supply of the branches of the celiac arteries. The main artery of the tumour blood supply was superselected by using a 2.9F microcatheter and a 2.7F microwire (Progreat, Terumo Medical Corp, Torkyo, Japan) depending on the tumor site; for example, the catheter was inserted into the left gastric artery for cancer of the upper and central stomach, and through the hepatic and gastroduodenal arteries into the right gastroepiploic artery for cancer of the lower part of the stomach (13). Oxaliplatin (100 mg/m^2^) and docetaxel (50 mg/m^2^) were used as arterial chemotherapy, and lipiodol (10 ml) mixed with oxaliplatin (2 ml) was used as embolic. Oxaliplatin, docetaxel and lipiodol mixed with oxaliplatin injected sequentially, and the injection time exceeded 5 min. When the mixture of lipiodol and oxalipatin was found to completely deposit in the tumour area and reflux to other blood vessels, the intervention was ended. After the intervention, the femoral artery was pressed for 15 min (Figure 1).

And the post-embolization syndrome, such as fever, abdominal pain, renal insufficiency, bleeding or gastric perforation after the GTC was carefully observed.

### Following chemotherapy

Three cycles of neoadjuvant DOS chemotherapy was performed every 3 weeks in this study. GTC was used as a first cycle of neoadjuvant chemotherapy (14). The second and the third cycle involved oxaliplatin (100 mg/m^2^) and docetaxel (50 mg/m^2^) were administered intravenously on the first day, and S-1 (40 mg/m^2^) was orally taken from day 1 to day 14. If the patients could not orally take S-1, 5-FU was administered intravenously instead.

### Surgery

After three cycles of chemotherapy, patients underwent gastroscopy and CT examinations to assess whether gastrectomy could be performed by two gastrointestinal surgeons with more than 10 years of experience. Patients were considered inoperable if they meet the following manifestations: (1) locally advanced cancer, including mesenteric root or para-abdominal lymph node metastasis that was highly suspected by imaging or biopsy confirmed, (2) lymph node invaded or surrounded large blood vessels (except for spleen artery), (3) gastric cancer with distant metastasis, and (4) tumor invasion of surrounding organs, extensive adhesions, and tumor fixation which presented technically unresectable.

### Definitions

This study assessed effectiveness based on the following indicators: (1) technical success, (2) clinical success, (3) complications, (4) pre-intervention and post-intervention obstruction remission, and (5) survival. The technical success of GTC referred to the successful selection of tumour-nourishing blood vessels and injection of chemotherapy drugs and embolic agents. Clinical success was defined as the score of GOOSS becoming higher above 2 after the intervention.

The time point at which the GOOSS was evaluate were before and 1 week after intervention. The scores were defined as follows: 0 no oral intake, 1 liquid only, 2 soft solids, and 3 complete or full diet (15). The Eastern Cooperative Oncology Group (ECOG) performance status (PS) was defined as follows: 0, normal activity; 1, able to walk freely and engage in light physical activities, but not heavy physical activities; 2, able to move freely and take care of themselves but have lost the ability to work, and are only able to participate in activities for no less than half of the wake time; 3, only able to partially perform self-care, and a bed or wheelchair is used for more than half of the wake time; and 4, completely bedridden (16).

The length of hospital stay is defined as the time from the start of the GTC to the discharge or the death of the patient. Complications were designated as intraoperative complications, bleeding, perforation, and they were classified according to Clavien-Dindo classification (17). Overall survival was defined as the time from intervention to death or the end of the study if the patient was still alive.

### Statistical analysis

Categorical and continuous data are presented as proportions, medians, and means and standard deviation, depending on the distribution. Independent-sample t test and Mann–Whitney *U* test were used to compare the indicators before and after the intervention. The Kaplan–Meier test was used to analyse the relationship between preoperative factors and overall survival (OS). The Cox proportional hazard model was used for univariate analyses. All statistical analyses were performed using SPSS 22.0 software. Tests of the hypothesis were statistically significant when the *p* value of a two-sided test was <0.05.

## Results

### Patient characteristics

Forty-two patients were included in our study, including 36 males and 6 females. The median age was 67 years. In terms of the ECOG, 22 patients received a score of 0, 8 patients received a score of 1, and 12 patients received a score of 2. Forty patients were diagnosed with clinical stage III and 2 patients were diagnosed with stage IV gastric cancer. Among all patients, 39 were diagnosed with adenocarcinoma and 3 were diagnosed with signet ring cell carcinoma. The baseline information for the BMI and albumin, haemoglobin, CEA, and AFP levels were shown in Table 1.

**Table 1:** Characteristics of patients.

Characteristics	No. 42
Sex (male/female)	36 (85.7%)/6 (14.3%)
Age (year) (median)	67 (35–84)
BMI (kg/m^2^) (median)	20.7 (15.4–30.4)
Albumin (g/L) (mean, SD)	35.1 (±5.9)
Hemoglobin (g/L) (mean, SD)	107.6 (±28.9)
CEA (ng/ml) (median)	2.4 (0.4–122.1)
AFP (ng/ml) (median)	2.6 (0.2–33.6)
ECOG (0/1/2)	22 (52.4%)/8 (19.0%)/12 (28.6%)
GOOSS before intervention (0/1)	8 (19.0%)/34 (81.0%)
**TNM stage**	
III	40 (95.2%)
IV	2 (4.8%)
**Histopathology**	
Adenocarcinoma	39 (92.9%)
Signet ring cancer	3 (7.1%)

Note: Variables are expressed as the mean ± SD, median or *n* (%).

BMI, body mass index; CEA: carcinoembryonic antigen; AFP, *α*-fetoprotein; ECOG, the eastern cooperative oncology group; GOOSS, gastric outlet obstruction scoring system; TNM, tumor-node-metastasis.

### Efficacy and complications

All of the 42 patients achieved technical success and 22 (52.4%) achieved clinical success. The median time of GTC was 83 min and the duration of hospitalization after GTC was 3 days. One patient experienced abdominal pain during and after GTC, after 5 days of conservation, the abdominal pain relieved. Twenty of the 42 patients underwent gastrectomy after intervention. Albumin levels, haemoglobin levels and GOOSS after intervention were shown in Table 2.

**Table 2:** Efficacy and complications.

Characteristics	No. 42
Technical success, *n* (%)	42 (100%)
Clinical success, *n* (%)	22 (52.4%)
intervention time (minutes) (median)	83.0 (30.0–180.0)
Albumin after intervention (mean, SD)	35.8 (±5.8)
Hemoglobin after intervention (mean, SD)	104.6 (±19.7)
GOOSS after intervention (0/1/2/3)	9 (21.4%)/11 (26.2%)/19 (45.3%)/3 (7.1%)
Median hospital stay (day)	3 (1–6)
Intervention-related complications, *n* (%)	1 (2.4%)
Gastrectomy after intervention, *n* (%)	20 (47.6%)

Note: Variables are expressed as the mean ± SD, median or *n* (%).

GOOSS, Gastric outlet obstruction scoring system.

### Comparison before and after intervention

Albumin levels, haemoglobin levels, and GOOSS measured before and 1 week after GTC were compared. Among the 42 patients, the preoperative score was 1 (0–1) and the postoperative score was 2 (0–3), which were significantly different (*p* = 0.000 < 0.05). No significant differences in albumin and haemoglobin levels were observed (*p* > 0.05) (Table 3).

**Table 3:** Comparison before and after intervention.

	Before	After	*p*
Albumin (g/L) (mean, SD)	35.1 (5.9)	35.8 (5.8)	0.609
Hemoglobin (g/L) (mean, SD)	107.6 (28.9)	104.6 (19.7)	0.630
GOOSS (median)	1 (0–1)	2 (0–3)	0.000*

GOOSS, Gastric outlet obstruction scoring system.

Note: Variables are expressed as the mean ± SD or median *p-value <0.05.

### Treatment and prognosis

**Figure 2:**
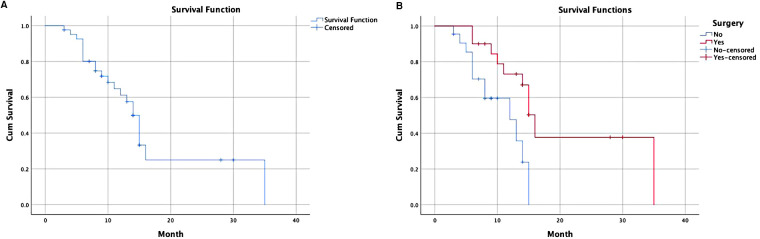
Survival analysis of patients underwent GTC. (**A**) A Kaplan–Meier overall survival curve of all patents underwent GTC; (**B**) subgroup analysis of surgical treatment group compared with non-surgical treatment group. GTC, gastric transcatheter chemoembolization.

Among all patients, the median follow-up time was 9.5 (3–35) months, and the overall survival time was 14 months (Figure 2A). No differences in sex, age, ECOG score, GOOSS and other indicators (*p* > 0.05) were identified in the multivariate survival analysis. A difference was observed between patients treated with or without radical gastrectomy after GTC (*p* = 0.014 < 0.05) (Table 4). Compared with the non-surgical group, the median survival time of the surgical group was 16 months, and the overall survival time of the non-surgical group was 14 months (Figure 2B).

**Table 4:** Prognostic factors for overall survival.

Variables	Univariate analysis		
	HR	95%CI	*p*
Sex (male/female)	1.608	0.585–4.418	0.357
Age (>/≤65 years)	1.512	0.606–3.772	0.376
ECOG (0/1,2)	2.167	0.849–5.529	0.106
BMI (>/≤20.7)	0.951	0.400–2.261	0.909
Albumin before intervention (>/≤35.1 g/L)	0.827	0.340–2.008	0.674
Hemoglobin before intervention (>/≤107.6 g/L)	0.626	0.257–1.527	0.303
GOOSS before intervention (0,1)	2.374	0.901–6.252	0.080
Surgery after intervention (yes/no)	0.304	0.117–0.786	0.014*

CI, confidence interval; HR, hazard ratio; ECOG, the eastern cooperative oncology group; BMI, body mass index; GOOSS, gastric outlet obstruction scoring system.

Note: *p-value <0.05.

## Discussion

In this study, the GOOSS was significantly improved in patients after GTC treatment. In a multivariate analysis, radical gastrectomy for gastric cancer was an independent prognostic factor for survival after GTC.

Gastric obstruction usually accompanies with advanced gastric cancer, and patients exhibit a limited survival. For patients with malignant gastric obstruction, the lack of an effective intervention will accelerate the progressive deterioration of the disease and death (18). Bypass surgery was the standard treatment option in the past, but it has many complications and a short survival time. Endoscopic stent placement is another treatment that reduces the incidence and mortality of malignant obstruction (19). Therefore, the best treatment for each patient must be chosen. A report comparing endoscopic stent placement with GJJ showed better short-term outcomes for stent placement (6). For GJJ, the long-term outcomes in terms of food intake, recurrent obstruction, and reintervention are better than other methods. In general, GJJ and endoscopic stents have their own advantages. Complications of endoscopic stents include stent obstruction, stent displacement, bleeding, stent rupture, perforation (8). The most frequent complications are stent obstruction and migration. Many complications have been observed after GJJ, such as an unsatisfactory improvement in oral eating, duodenal reflux, and vomiting of bile fluid.

The purpose of palliative care is to relieve obstruction-related symptoms and improve the quality of life of patients. GTC is not only a palliative treatment but also a conversion treatment. The goal of GTC is to convert unresectable gastric cancer with obstruction into resectable gastric cancer to achieve R0 radical surgery. In the present study, patients who underwent total gastric cancer resection after GTC experienced a significant advantage over those patients with a poor response to chemotherapy after GTC.

The current technical approach used to treat gastric cancer with obstrution is to bypass the tumour using GJJ or place a stent to spread the tumour and locate a pathway for food to be transported (20). This study provides a new approach designed to eliminate the tumour that caused the obstruction and allow the food pass smoothly. This approach also coincides with the successful application of GTC, which improves patient survival. In addition, a surgical method such as GTC may allow surgeons to perform another interventional operation, GJJ, or stent implantation if an obstruction recurs. Thus, GTC provides an opportunity for reoperation in patients with an obstruction.

Transcatheter arterial chemoembolization is commonly used in solid organs such as the liver and is considered a feasible and effective method for treating tumours (21, 22). Some case studies have reported the success of GTC combined with neoadjuvant chemotherapy. In the present study, the embolizing agent was the mixture of lipiodol and oxalipatin, and an intra-arterial infusion was administered during the GTC. Then, lipiodol and oxalipatin were injected to ensure drug deposition and tumour blood embolism. This method allows the local tumour to undergo necrosis without causing gastric perforation. Gastric perforation did not occur in any of our patients. We consider this method of local administration and embolization safe. This approach represents an interventional treatment strategy for patients with gastric cancer with obstruction, and provides patients the opportunity to undergo secondary surgery.

In recent years, chemotherapy has become an important treatment for advanced cancer because it prolongs the overall survival and provides patients a better quality of life. For patients with gastric cancer, the intensity of symptoms and status before treatment are related to survival rate. In addition, chemotherapy has been shown to improve the survival of patients with gastric cancer (23–25). In this study, patients who underwent GTC routinely recommended another two cycles of chemotherapy and showed a survival benefit. GTC combined with chemotherapy may provide patients with surgical opportunities and prolong the survival time by alleviating the obstruction.

There are also some key suggestions for the GTC process. Because the blood supply of the tumor is abundant, each artery should be carefully observed during celiac arteriography. For elderly patients, the arteries are tortuous, and there may be atherosclerotic plaques at the openings of arteries, these can sometimes make it difficult for the microwires to be inserted. In these cases, the guidewires should be rotated slowly to superselect to the tumor-nourishing blood vessels. Furthermore, the entire time of drug administration should be slow and more than 5 min, after that, the mixture of lipiodol and oxalipatin was admistrated to deposit the chemotherapeutic drugs and embolize the blood vessels.

This study has certain limitations. First, this was not randomized but was a retrospective analysis. Second, the lack of an evaluation of the quality of life is the main limitation of this study. In the future, the design must be standardized to assess related issues, such as the quality of life. Furthermore, the small number of patients and samples included, and the short follow-up time also affected the accuracy of the study.

In conclusion, GTC is a safe and effective treatment, and furthermore, it could be an alternative method in treating advanced gastric cancer with obstruction. Patients who have the opportunity to undergo radical gastrectomy after GTC will experience a survival benefit.

## Data Availability

The raw data supporting the conclusions of this article will be made available by the authors, without undue reservation.
